# Research on the Public Opinion Guidance Mechanism of Major Public Health Incidents

**DOI:** 10.3389/fpsyg.2022.872464

**Published:** 2022-06-29

**Authors:** Yuqi Wang, Rui Wu, Jun Zeng, Peiyi Xue

**Affiliations:** ^1^School of Humanities, Jiangxi University of Finance and Economics, Nanchang, China; ^2^Jiangxi Institute of Fashion Technology, Nanchang, China

**Keywords:** major public health incidents, public opinion analysis, guidance mechanism, network text analysis method, COVID-19 pandemic

## Abstract

Public opinion guidance plays a crucial role in the management of major public health incidents, and thus, exploring its mechanism is conducive to the comprehensive governance of social security. This study conducts a case study on the anti-pandemic public opinion guidance and analyzes the public opinion representation and the internal mechanism of public opinion guidance in the context of the COVID-19 in China. The findings suggest that the public opinion on the COVID-19 manifested a three-stage progressive and stable tendency and witnessed the strength of China, specifically, benefiting from the systematic and complete integration and release mechanism for anti-pandemic information, the three-dimensional mechanism for the dissemination of knowledge related to pandemic prevention and health, the innovative disclosure mechanism for precise information, and diversified channels for international public opinion guidance. The guidance mechanism proposed in this study provides significant suggestions for the public opinion guidance of global major public health incidents in future.

## Introduction

In recent years, global major public health incidents have occurred frequently, and those characteristics, such as suddenness, unpredictability, and disruption of social stability, have attracted widespread attention from both scholars and practitioners (Xiao et al., 2021; Zhang, 2021). Liu (2004) argued that the public health crisis like SARS exposed the fragility of the public health system in China, making it difficult to respond to public health emergencies promptly and coordinately. Mahony and Clarke (2013) analyze the contemporary context of public crises, calling on society to give more attention and support facilities to public governance. With the outbreak of the COVID-19, scholars in different fields and countries put more attention on the governance of public health incidents (Ortega and Orsini, 2020; Shin, 2021; Yuan, 2021). A series of research has been carried out to governor global public health emergencies, including the response, emergency management capabilities, and assessment measures (Stenseth et al., 2021), on the theoretical improvement and path design of contingency plans for major public health incidents (Mullen et al., 2020), on the perfection of the theoretical system of monitoring and early warning of public health emergencies (Bennett and Carney, 2017), and the theory and construction mechanism of crisis management of public health emergencies (Deitchman, 2013; Hu et al., 2018). In general, the academic community has reached a consensus on emergency management measures for public health incidents. It is urgent to proceed from the stages of reduction, readiness, response, and recovery (4R model), and all the stages shall be complementary to each other for the public governance through an evolutionary mechanism. Under the evolution of major public health incidents undergoing “germination, outbreak, stalemate, and recession,” there are traces of their evolving regularities at all stages (Hu et al., 2018). Therefore, it will be more rational and effective to analyze the governance of major public health incidents from the perspective of changes at different stages.

With the outbreak of COVID-19 at the end of 2019, people are often in a state of high tension due to fear of virus infection, and the demands for understanding relevant public opinion information explode. If the government fails to act promptly to convey crisis information to the public effectively and efficiently, it will inevitably cause the public to be panic and anxious in the case of a public health crisis (Chen et al., 2020). At the same time, the Internet has become a key channel for the masses to understand the progress of epidemic prevention and control and information related to the epidemic situation (Milenkovic et al., 2020). Without reasonable, scientific, and effective public opinion guidance and governance, public opinion may evolve into information public opinion, and the crisis caused by it is equivalent to the threat and panic of a major epidemic situation, which is serious damage to public security and social stability. Particularly in social media, due to the large number of participants involved in the information dissemination and discussion on pandemic-related matters, a multi-party public opinion space has been formed.

Consequently, the correct guidance of public opinion plays a pivotal role in pandemic prevention and control. Public opinion guidance must correctly disseminate information and values to make the public opinion fully expressed and effectively interacted under a reasonable premise. This is a dynamic balance mechanism to maintain and promote the benign operation of public opinion. In the post-pandemic period, public opinion guidance is playing an increasingly prominent role in major public health incidents in China.

Based on the above background, this study will combine previous research on crisis communication and public opinion guidance with case studies of recent outbreaks to explore the evolution of public opinion at different stages, so as to provide operable practice models and ideas for public opinion guidance.

Social media have been rapidly emerging in recent decades. As a virtual community based on information sharing, social media have become a major platform for information dissemination and opinion exchange by increasing user engagement through satisfaction and positive emotions. The production and interpretation of online text production are not only influenced by “individual communicative nuance” (ICN), but also constrained by the complex power relations and environmental factors behind it (Kelsey and Bennett, 2014). As with many virtual spaces, social media have its own rules of discourse, and as society evolves, the main users of each platform are constantly refining their discourse system, and social media are no exception. Studies have shown that Weibo users flexibly use indirect discourse strategies to express their emotions and attitudes (Tao, 2021) to increase the visibility of their discourse and secure their own discursive space.

Despite the over-surveillance in social media, current media algorithms are becoming more sophisticated and the weight of information is not based on a single indicator, but rather on a variety of “retweets,” “comments,” “shares,” and other indicators, coupled with the construction and continuous improvement of each platform’s discourse system, users can use a variety of ways to express their views and opinions (Hwong et al., 2017). In the case of social events, the excessive surveillance of social media does not obscure users’ statements. Therefore, this study concludes that it is reasonable to choose Weibo as the data source.

## Literature Review

### Research on Crisis Communication

The public crisis is triggered by natural disasters, failures of social operation mechanisms in the process of social operation, and has a low probability of occurrence (Waugh, 2003), but it can seriously affect the image and authority of the government or related organizations (Pearson and Clair, 1998; Lerbinger, 2012). In the era of big data and individual actual position availability, researchers scientifically grasp individual behavior under multiple contexts, such as retailing (Li and Katsumata, 2020; Li et al., 2021a,b). Specifically, online social media are a crucial window for individuals to obtain information and express their emotions (Sun et al., 2017; Li et al., 2018; Wang et al., 2020, 2022). The particularity of public health emergencies makes the online public opinion continuously heat up. Accordingly, the academic community has carried out a detailed exploration of crisis communication. Specifically, it mainly presents two research directions.

One focus is put on the performance characteristics of public relations to primarily explore whether the crisis communication strategies adopted by competent agencies are reasonable and effective when a crisis occurs and how the public responds to the organizational crisis (Russell Neuman et al., 2014). Under this orientation, Barton (1993) proposed the five-link model of crisis management, including detection, prevention, containment, recovery, and reflection. However, in most cases, the organizations do not cope with crises alone all the time. Sometimes, when the public freely and openly defend the organization, they become active participants in crisis communication (Schoofs and Claeys, 2021). Also, some scholars have studied and analyzed the message transmission of crisis information on a company’s website by drawing on the concept of co-creation of meaning from the situational crisis communication theory and summarized the characteristics of public opinion communication of emergencies (Haupt, 2021; Waters and D’Urso, 2021). The other focus is on the management and restructuring of the organizational image after crises. Relevant studies suggested that after a crisis incident, the damage to related organizations and institutions results from many sources, but the most direct and fatal damage is the loss of the organization’s reputation and image (Lee and Jahng, 2020). Therefore, the emphasis of such research is mostly put on the strategies and path design for the organizations to resolve the crisis and restore their image after the crisis (Bradford and Garrett, 1995; Su et al., 2021). To promote the modernization of the social governance system and governance capacity, new directions and new propositions should be put forward regarding the public opinion guidance mechanism (Zhang et al., 2020; Ye, 2021).

Previous studies have shown that the image restoration strategies for organizations in crisis incidents are cumbersome, and it is urgent and crucial to apply pertinent theories to systematically guide the practical research of crisis communication (Triantafillidou and Yannas, 2020; Kochigina et al., 2021). Most of the existing studies have explored crisis communication strategies from an abstract perspective. For example, some scholars combined the crisis situation with crisis response strategies in their research, emphasizing the options of crisis response strategies and methods of communicating with the public in the process of organizational communication and image reconstruction in different contexts of the crisis communication (Johansson and Odén, 2018; Liu et al., 2021). Some scholars especially emphasized the influence of the organization on the choice of crisis handling strategies, that is, the sense of responsibility in the crisis, believing that the relationship between crisis situations and crisis strategies should be mediated through crisis responsibility (Kiambi and Shafer, 2016; Nekmat and Kong, 2019; Xu, 2020). For instance, the organization’s choice of crisis management strategies will be affected by the public perception of crisis incidents, responsibility, and other attributors (Xu and Wu, 2020).

In major public health incidents, when the public faces the situation of “missing information” or “surging desire to know information,” the traditional information disclosure channels are no longer able to meet the needs of the public (Yong et al., 2020). In this specific situation, social media, in the form of information sharing, play a role in filling the lack of information and guiding the trend of public opinion during special periods (Kwok et al., 2021). From the perspective of crisis situations and crisis strategies, this study analyzed the crisis situation of public speech and rumors on Sina Weibo (microblog) to explore how the media in the face of major public health emergencies adopt appropriate mechanisms and strategies in accordance with the crisis situation.

### Research on Public Opinion Guidance

In the traditional research on public opinion, scholars focused more on the description and interpretation of public opinion at a single point of time. Such research defined public opinion as “relatively unanimous opinion” and mostly conducted public opinion surveys to collect and analyze personal opinions expressed by individuals and generalize them into public opinion at the aggregate level (Gao, 2021). However, most of these studies presented a static view of public opinion, ignoring the essence of the dynamic fermentation and evolution of public opinion in the time dimension (Huang et al., 2018). Arguing that the public behavior changes in response to the public opinion are of non-linearity, some research proposed that the development of “issue and attention” can be analyzed in five stages, including the pre-issue stage, the detecting and early warning stage, the cost reflection stage, the fading stage of interest in issues, and the post-issue stage (Zhao, 2017). In addition, some research on public opinion suggested that in the event of major public health emergencies, the attention of traditional media increases as the public attention increases. The initial attention is generally low, and only as the popularity increases, the degree of coverage be gradually enhanced (Ting, 2020). After the continuous fermentation of social public opinion, the government media and social media release relevant information on Weibo to realize the guiding role of public opinion and ensure a favorable developing momentum of public opinion (Ai et al., 2018; Chen, 2020).

In discussing the timing of emergency and public opinion guidance, Liu et al. (2022) put forward a multi-stage risk grading model of Internet public opinion in research on COVID-19. This model effectively focuses and monitors the risk degree of public opinion to public health emergencies. More scholars’ findings imply that public opinion is formed earlier than crises actually materialize (Asano et al., 2021). When social media becomes the “main forum” for public discussion, it is crucial to understand the public’s psychological state and provide inspiration for communicators to conduct positive public opinion guidance (Chiang et al., 2021; Yi et al., 2022).

Based on the research results of the above literature, this study applied the 4R model to divide and analyze the staged development of major health practices and relevant discussions on the roles of all sectors of the society during the pandemic. To conduct a comprehensive study of the participants in the online public opinion information, this study selected several types of users from the official news media, medical institutions, and netizens as the research object to explore the evolving process of opinions on the topic of “COVID-19.” Besides, it explored the popularity of public opinion in different periods through the high-frequency words that people used in the discussion during the pandemic. Finally, it analyzed the people’s psychological state changes after receiving relevant public opinion guidance given the rapid information changes in the new media era.

## Case Selection and Public Opinion Analysis

### Case Description of Anti-COVID-19 Public Opinion

After the outbreak of the COVID-19 virus at the end of 2019, China took strong measures to fight against the pandemic and achieved major achievements in a short period of time. Specifically, in the early days of anti-pandemic in China, people fell into confusion and panic in the face of the unprecedented major public health incident. From being a top searched hashtag on 31 December 2019, until 20 January 2020, the public opinion on the COVID-19 was in its infancy. Although the pandemic topic showed signs of rising among varied topics, it did not attract people’s great attention. Later, during the period from 20 January 2020 to 20 February 2020, as the Spring Festival travel rush started and the population mobility increased, the pandemic spread rapidly and the panic buying and shortage of masks began to appear. The information dissemination of the pandemic entered a new stage, and after a few months, it became crucial information that people scrambled to communicate and transmit. Information in different forms and on various platforms emerged frequently, public opinion was in an uproar, the experts refuted rumors, public information on governmental portals continued to infiltrate, and online public opinion gradually changed from blind suspicion to rational understanding. Finally, starting from 21 February 2020, after a period of fermentation of the public opinion, the crisis communication gradually stabilized. Major online media came to the second stage of public opinion guidance, and the work of refuting rumors and appeasement was put on the agenda. During this period, the sudden surge or occurrence of pandemic-related incidents in a certain place became the main theme of public opinion, and the fluctuations of people’s emotions gradually became stable.

### Data Collection

This study used Octopus software to collect online comments of the COVID-19 as the samples from the target website Sina Weibo, the largest online social media in China. The reason for choosing Weibo is that it is a network platform for a large number of governmental agencies, celebrities, and news media to release information. With openness and public attribute, Weibo is the “main battlefield” for the interaction between the transmitters and the audience, and its transmission capacity is far more than that of traditional media, enabling people from all walks of life to break the boundaries of physical space and express their opinions on public issues. Therefore, the online data collection from Weibo is of significant scientific nature and validity for the study of the public opinion guidance mechanism of major public health incidents.

The data collected in the study dated from 27 December 2019 to July 2021. Under the theoretical framework of the 4R model, the developing trend of public opinion on the pandemic was divided into three stages: the initial fermentation stage started from 27 December 2019 to 19 January 2020, the mid-term uproar stage dated from 20 January 2020, to 20 February 2020, and the late continuation stage was from 21 February 2020 to July 2021. The total number of samples collected in this study was 22,700. ROST CM6 was used to conduct high-frequency vocabulary, network semantics, and sentiment analysis to effectively analyze the development and guidance direction of the public opinion under the pandemic, providing a typical case for the study of public opinion guidance mechanism of major public health incidents.

ROST CM6, as content analysis software, is based on the principle of machine learning with human assistance, which can better distinguish word emotions in Chinese contexts and automatically generate analysis results. It can automatically generate analysis results into three categories: “positive sentiment results,” “negative sentiment results,” and “neutral sentiment results,” which are widely used in Chinese studies.

### Research Findings

From the perspectives of high-frequency vocabulary, network semantics, and sentiment features, this study, respectively, explored the sample presentation rules of the three stages of the public opinion, including the initial fermentation stage, the mid-term uproar stage, and the late continuation stage.

**FIGURE 1:**
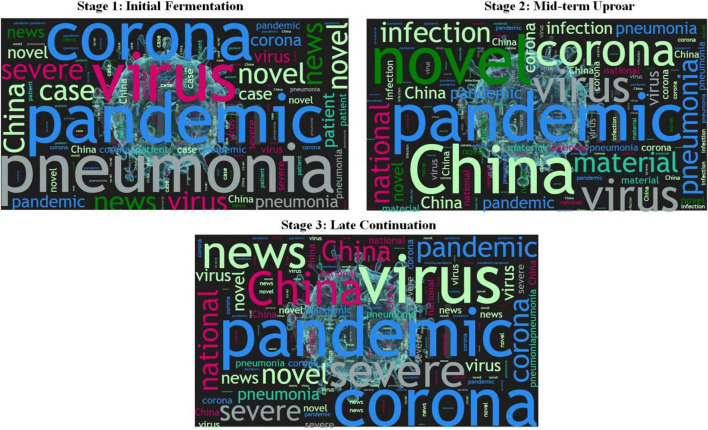
High-frequency keywords in each stage.

In terms of high-frequency vocabulary analysis (as shown in Figure 1), the keywords of the first stage consisted of pandemic, virus, pneumonia, coronavirus, novel, news, case, severe, patient, and China. The keywords of the second stage included pandemic, microblogging, virus, pneumonia, China, novel, coronavirus, national, material, and infection. The keywords of the third stage comprised pandemic, China, severe, coronavirus, virus, microblogging, novel, national, pneumonia, and news. From the evolution of word frequency, it indicated that the COVID-19 gradually drew people’s attention and concern after Wuhan officially announced lockdown of the city. Public opinion in the first stage put primary emphasis on the “COVID-19 outbreak”; in the second stage, it gradually turned to demand microblogging and the national image of external communication began to be noted by the people; in the third stage, more emphasis was given to the national image promotion, and phrases like “made in China” and “China’s strength” ranked top gradually.

**FIGURE 2:**
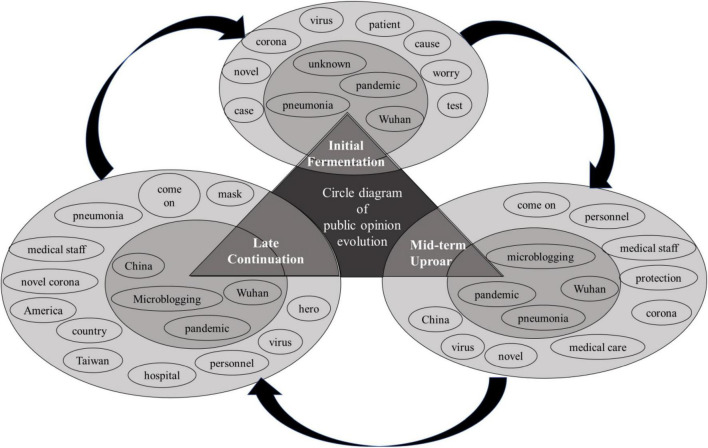
Network semantic relations of keywords of the public opinion in major public health incidents.

In terms of network semantic analysis (as shown in Figure 2), the central circle in the first stage included “pandemic, unknown, pneumonia, Wuhan,” and the second circle consisted of “patient, case, corona, novel, virus, test, cause, worry”; the central circle in the second stage comprised “Wuhan, pandemic, microblogging, pneumonia,” and the second circle included “personnel, medical staff, protection, come on, medical care, corona, novel, virus, China,” and of these, the centrality of “Wuhan” was relatively high; in the third stage, the central circle consisted of “Wuhan, pandemic, China, microblogging,” and the third circle included “pneumonia, novel corona, virus, medical staff, personnel, hospital, Taiwan, the United States, hero, country, come on, mask,” and its specific dissemination mechanism was shown in Figure 2. It suggested that under the pandemic, the public opinion moved from initially having no idea to making clear of the source and then to having divergent in-depth discussion on different topics. The three-stage network semantic analysis further demonstrated the conclusion drawn from the high-frequency vocabulary analysis, that is, the central circle had significant features.

The difference is that in the first stage, the lack of understanding of the epidemic has become part of public opinion, and in the face of the new unknown disease, public opinion has shown great concern for seeking medical explanations. There are “unknown” in the initial core circle, “new”, “virus”, “test”, “reason”, “worry”, etc. in the secondary circle. At the second stage of public opinion fermentation, “unknown” was no longer the core of public opinion, and “microblogging” quietly entered the core circle. The departure of adjectives and the entry of nouns indicate that user sentiment is moving in a stable direction under the big data, so words like “lucky” and “protection” and “medical care” are beginning to appear in the secondary circle. The words “protection” and “medical care” also show that public opinion is beginning to pay more attention to what can be done. At the third stage, the core of public opinion has all changed into nouns, and the names of other countries and regions have appeared in the secondary circles because of the development of the epidemic. It can be seen that the number of words in the secondary circles has also increased significantly, which indicates that public opinion at this stage radiates outward and participants begin to pay more attention to non-local information about the epidemic, reflecting the pluralism of public opinion and the smoothness of public sentiment.

**FIGURE 3:**
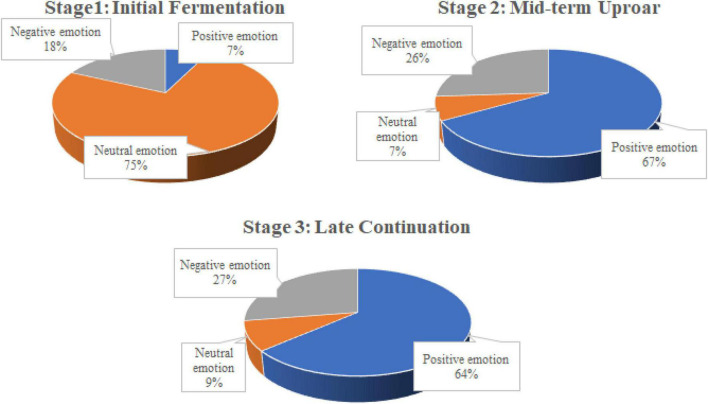
Emotional distribution of the public opinion at each stage.

In terms of sentiment feature analysis (as shown in Figure 3), in the first stage, neutral emotion accounted for 74.54%, occupying a dominant position, and negative emotion accounted for 18.08%, mostly at a high level, while positive emotion accounted for 7.38%, mostly at a low level; in the second stage, positive emotion accounted for 66.79%, dominant, showing a slightly lower level, and neutral emotion stood at 7.16%, while negative emotion stood at 26.05%, showing a low level; in the third stage, positive emotion reached 63.6%, dominant, showing slightly low-to-medium level, and neutral emotion accounted for 8.89%, while negative emotion stood at 27.51%, showing a low level.

The change in user sentiment was highly correlated with the point of development of the COVID-19 outbreak, and the presence of two experts in the medical field at a high-level government meeting on 20 January 2020, provided a “reassurance” to the more negative public opinion in the previous period. Six days later, the first square-cabin hospital in China was put into operation and delivered 13 days later. The professional interpretation of the experts and the efficient action of the government became the key to the change of public sentiment in a positive direction. What can be seen is that in the second phase of public opinion around the new outbreak, the “unknown” in the core vocabulary and “worry” in the sub-circle have disappeared, replaced by medical-related words, such as “medical,” “protection,” “medical,” and “good luck,” and public opinion has started to turn positive. This is consistent with the results of public opinion analysis by ROST CM6 software. In the late stage of public opinion, “Wuhan” and “epidemic” are still the focus of public opinion, but other countries and regions start to appear in the sub-circle, indicating that at this stage, the focus of Chinese public opinion starts to extend outward, and China’s medical and material assistance to other countries and regions becomes a more popular topic. This behavior was also seen as a positive sign that the development of the epidemic in China was manageable at the time, so it is not surprising that public sentiment continued to remain positive during the third phase.

In general, the ups and downs of emotion in the three stages were mainly the process of neutral emotion in the first stage to positive emotion in the second stage, and the emotional expressions in the second stage were basically at the same level as the third stage. In the early stage when the public acquired the information on the novel coronavirus, most people did not have direct emotional changes, and the majority emotional change was negative emotion, while in the mid-term and late stages of the public opinion fermentation, people were in a stable positive emotion, and they were more confident in the effective prevention and control of the COVID-19.

## Research Findings and Guidance Mechanism Analysis

Based on the high-frequency vocabulary and emotion analysis of the obtained texts, and in combination with the content of news reports in corresponding periods, we summarized the evolution law of public opinion in different stages of COVID-19 and the emotional feedback of reports, to observe how the release and dissemination of information affect the expression of public opinion in the media. Overall, during the anti-pandemic period, apart from the public’s incomprehension of relevant information and concepts in the initial stage, the public outcry did not show a chaotic situation in the second stage; instead, the specific characteristics of the pandemic have been clarified and the positive confidence in governance has been initially established, reflecting strong monitoring and governance capabilities of public opinion, which has a relatively complete public opinion guidance mechanism. Taking account of the existing information of public opinion guidance, it is clear that the current situation of public opinion control is inseparable from the established anti-pandemic information integration and release mechanism, the three-dimensional mechanism for the dissemination of knowledge related to pandemic prevention and hygiene, the innovative precise information disclosure mechanism, and the diversified channels for the international public opinion guidance.

### Integration and Release of Anti-pandemic Information

The analysis of high-frequency vocabulary suggested that the keywords, such as pandemic, virus, pneumonia, corona, novel, and China, maintained the top 10 ranking in the 3 stages, indicating that the spread of anti-pandemic information had a stable effect, and accurate crisis information was released immediately at the beginning. As a result, it is inferred that China has established a systematic mechanism for information integration and release so that information related to the COVID-19 is released in a timely and accurate manner. Specifically, a systematic and sound anti-pandemic information integration and release mechanism include the following three respects.

First, the working group of anti-pandemic public opinion guidance is timely established. This is necessary for information coordination to achieve good public opinion concerns. These working groups include the “Information Promotion Group” responsible for news media reporting and regulating the release of information. In addition, other working groups have also formed “public opinion information groups” to undertake the collecting and sorting out of the information in the field to improve the pertinence of their work. Through the information release of the working group, lead the public’s attention to public health emergencies and form a public agenda. This is consistent with the focus of public discussions during the evolution of the epidemic, that is, the results of high-frequency vocabulary analysis.

Second, the mainstream news media should release information timely and accurately. After Wuhan was locked down on 20 January 2020, a variety of mainstream news media at all levels released information related to the pandemic in a timely and accurate manner. For example, *People’s Daily*, its major news page continuously increased the anti-pandemic information coverage, and some pages were even exclusive for anti-pandemic coverage from 21 January 2020 to 22 February 2020. In addition to the anti-pandemic information covered by the national mainstream media, local mainstream media also released relevant news to timely and accurately inform the public of the local anti-pandemic situation. These reports were beneficial to the public to have a comprehensive understanding of the national and local anti-pandemic situation.

Third, the micro-media of governmental departments should assist in supplementing relevant information. Besides the news reports of the mainstream news media, governmental agencies at all levels also used their own micro-media like WeChat official account and Weibo to assist in the anti-pandemic public opinion guidance. The innovative reporting method has also aroused great repercussions and attention across the country. Judging from the content of the information released, it introduced the basic situation of the pandemic, the overall countermeasures, major achievements, and hygiene knowledge, timely and effectively supplementing the anti-pandemic information of the mainstream media and effectively guide the public opinion during the anti-pandemic period. This is critical for public information acquisition and stabilizing sentiment, as well as reflecting the organization’s crisis responsibility.

### Anti-pandemic and Hygiene Knowledge

The network semantic analysis indicated that the central circle of the three stages contains the two central points of “Wuhan” and “pandemic,” and “microblogging” and “China” are also in the central area or sub-circle, suggesting that around the central topic, the public opinion of the pandemic takes on three-dimensional presentation of knowledge dissemination, and the public obtains the core information in different periods, owning to the current three-dimensional dissemination mechanism of pandemic prevention and hygiene knowledge.

The purpose is to change the public’s attitude of not paying attention to the epidemic in the early stage. In the process of guiding anti-pandemic public opinion, governmental departments and the media adopted a three-dimensional dissemination mechanism for the pandemic prevention and hygiene knowledge to deepen the public’s awareness of the pandemic situation and better cooperate with the prevention work. The three-dimensional mechanism is mainly manifested in the following three aspects:

First, in terms of the contents, a detailed, multi-angle, multilevel analysis and popularization of relevant pandemic prevention and hygiene knowledge is conducted, such as the causes, transmission routes, harms, isolation, and precautionary measures, to enable the public to have a comprehensive and in-depth understanding of the COVID-19.

Second, in terms of the carrier, when introducing knowledge about pandemic prevention and hygiene, governmental departments use a variety of media channels and media technologies. A wider range of information push has been popularized, which ensures the guidance of public opinion in the process of public opinion fermentation and continuation.

Third, in terms of communication modes, information forms, such as text, image, video, and animation, are adopted to achieve a three-dimensional effect. In addition to text and image to popularize and explain how to prevent the COVID-19, it has formed a strong sensory stimulation to the visual and auditory systems of the public, inoculating them with a “vaccine” mentally to make them actively cooperate with preventive measures during the pandemic.

### Innovative Disclosure Mechanism of Precise Information

Confronting major public health incidents, the public may change their emotion with the acquisition of information. The analysis of public opinion suggested that in the context of the pandemic after the public experienced initial confusion and negativity, they showed firm confidence in the fight against the pandemic in the mid-term uproar stage and late continuation stage of public opinion. This is attributed to the disclosure and sharing of precise information.

The success of public opinion guidance depends on the disclosure and circulation of key information, and the precision of its publicity also has a great impact on the effect of public opinion guidance. During the pandemic prevention and control, there emerged new changes in the disclosure of relevant information, such as the activity trajectory of suspected cases like close contacts. The information disclosure has extended from *the city level* to the *community level*. The degree of information disclosure of previous major public health incidents was not high. The level of information disclosure is classified as *the city level*, and the cognition and understanding of information are still relatively vague.

In the context of COVID-19, the overall prevention requirements and citizens’ personal privacy were considered, and the information related to the pandemic was disclosed to *the community level*. By disclosing the suspected cases’ means of transport and taking account of the health code, it is also beneficial for real-time traceability to find and isolate the suspected passengers in time. When the information was disclosed to the community level, which enables risk areas to minimize the impact on the normal work and life of the public.

### Diversified Channels for International Public Opinion Guidance

In the analysis of public opinion, vocabulary, such as “the United States” and “country,” appeared in the conclusion of the sub-circle in the network semantic analysis of the third stage, and public discourse is turning toward the comparison between countries.

First, it is crucial to build a multilingual and multilevel interactive information dissemination network. A number of foreign language service hotlines for pandemic prevention and control have been opened in a timely manner to respond to the concerns of foreigners living in China. This is the embodiment of crisis responsibility in the face of public health emergencies. Making the public mood change because of the performance of the organization, behavior is the best embodiment.

Second, it is vital to strengthen the publicity and coverage of mainstream media. The mainstream media took the initiative to avail of their communication advantages to disseminate relevant information abroad in a targeted manner. *China Daily*, as a key window of international communication, launched the online English page “Measures to Combat the COVID-19 Outbreak.” Focusing on the anti-epidemic issues and information needs of the international community, the meeting released the latest progress in vaccine research and development and drug efficacy in the shortest possible time and shared confirmed and effective prevention and treatment measures. Authoritative information is the driving force for stabilizing public opinion.

In addition, a major feature of anti-pandemic public opinion guidance lies in the effective use of international academic exchanges to strengthen public opinion guidance. In April 2020, The Lancet, an authoritative British medical journal, published an academic paper jointly completed by Chinese and American scholars in its infectious disease monthly publication, titled “*Epidemiology and transmission of COVID-19 in 391 cases and 1286 of their close contacts in Shenzhen, China: a retrospective cohort study*,” which systematically introduced successful experience in containing the pandemic (Bi et al., 2020; Zou et al., 2020).

To sum up, the sound information integration and release, and the spread of knowledge about the COVID-19 outbreak, measures, such as establishing the system of information dissemination mechanism, which laid a foundation for public opinion guide. It is the release of systematic information and the setting of related topics that have turned the public’s attention from the beginning of the epidemic to the later positive attitude toward the epidemic. A large number of news reports have broadened the channels for international communication. While presenting the international epidemic situation to the public, they have also contributed China’s plans to the international community, strengthened public opinion guidance through international academic exchanges, and played a positive role in regulating public sentiment.

## Research Conclusion and Implications

### Theoretical Implications

This study adopted the 4R model to define the developing state of public opinion, emphasized the importance of different time stages in the research on public opinion communication guidance, and revealed that there exists a dynamic process that interrelates and interacts with each other between different entities in the development of the event, public opinion guidance, and public sentiment changes. It will also help to understand the synchronization and lag effect of public emotional reaction and related measures and news reports in public health emergencies. Moreover, it adopted the network text analysis method to explore the changes of public sentiment guided by public opinion during the pandemic. The findings revealed that the public’s emotional change in the direction of public opinion was consistent with the crisis management model proposed by Barton. Open information channels and effective information response make the public opinion guiding situation still manageable. This broadens the public’s cognition, attitude, behavior, and the final effect may change with the different stages of public opinion guidance.

Furthermore, this study analyzed the public opinion guidance mechanism of crises and demonstrated the efficacy of the public opinion guidance strategy in China, which highlighted the improvement of information disclosure mechanism. In the second stage of public opinion development, the Chinese government used the expertise of experts to provide theoretical endorsement to prove the controllability of the epidemic, the distant “victims” of the epidemic, and stabilize the emotions of the netizens at home who were under control due to the epidemic; at the same time, it actively took effective measures to carry out a series of medical rescues in Wuhan, so that the people of Wuhan could receive timely help and stabilize the emotions of the people in the eye of the storm. At the same time, core experts, on the one hand, carried out continuous observation and research on the virus and, on the other hand, responded to the core concerns of the masses in a timely manner, and medical and psychological researchers were also actively involved, using the Internet for effective knowledge and information popularization, supplemented with the publicity of anti-pandemic knowledge.

### Practical Implications

Based on the case analysis of the COVID-19, this study provides the following practical implications for the public opinion guidance of major public health incidents. First, our own public opinion position must be consolidated. The security of public opinion is an important component of national security; thus, the means of media shall be reasonably exerted to release information in line with the development trend of the times. Filling in the information gap between ordinary people and the media is the key to strengthen the guidance of public opinion and consolidate the position of public opinion. The media should keep in step with the government as a “bridge” between the government and the public to refute rumors in time and maintain the healthy operation of the network information ecology. Furthermore, when sensitive information is involved, it is necessary to strengthen the coordination and communication between the publicity department and the national security department to make strategic plans at the macrolevel and enhance the guidance of cooperation between different units at the microlevel in a bid to form a joint force that serves the national interests.

Second, the information must precisely match the needs of users. People first is the basic principle of public opinion guidance. This study revealed that all kinds of urgently needed information require the audience being a community of emotional imagination. This is not only more open and fully mobilizes the enthusiasm of the audience, but also highlights the real-time interaction of public opinion guidance. Taking into account that different groups have different access to information, the media should use different methods to transmit correct information and expand the coverage of public opinion guidance. In addition to presenting news objectively, the media should pay attention to the acceptance of the audience, accurately locate the recipients of the public opinion guidance, and diversify the reporting methods with an aim to give full play to their credibility, power, and guiding capacity to effectively disseminate objective facts.

Third, the media literacy of the public must be improved. The public should be cultivated to be able to use official media, understand official media, and rely on official media in the era of the Internet, instead of blindly following others’ opinions. During a sudden crisis, how to keep the public calm in the chaotic field of public opinion and how to make the official information accurately meet the needs of the public to effectively resolve questions and doubts are the key to guide the public opinion, ease the public’s negative emotions, and stabilize the society in the case of major public health practice. The mainstream media should give full play to their role in strengthening confidence, warming people’s hearts, and pooling people’s efforts to stand at the center of public opinion, grasp the trend of public opinion, and publish relevant information accurately and reasonably to build a harmonious and healthy public opinion ecology.

### Research Limitations and Future Direction

Under the framework of procedural public opinion analysis, this study analyzed the correlation between the public opinions on the COVID-19 outbreak on Sina Weibo and the public sentiments and discussed the popularity of pandemic-related topics and the effect of public opinion guidance in different stages. However, as the network text analysis mainly extracts opinions and remarks of the public regarding a certain matter in the network within a period, there are differences with regard to the releasers of the public opinion in addition to the quantity; accordingly, this study is of some subjectivity. Besides, since this study was divided into time periods during the development of COVID-19, it was impossible to observe the expression of people’s opinions at time points and their subsequent emotional changes guided by public opinion, for example, the emotional changes after different events and different time points. Since the social impact of pandemics is a dynamic process, in such a rapidly changing environment, our findings are limited, so the findings of this study need to be continuously improved by subsequent studies. Due to limited time and energy, this study is limited in its generalizability and application. Future research may consider the above-mentioned points for further exploration.

## Data Availability Statement

The original contributions presented in the study are included in the article/supplementary material, further inquiries can be directed to the corresponding author.

## Ethics Statement

Written informed consent was obtained from the individual(s) for the publication of any potentially identifiable images or data included in this article.

## Author Contributions

YW and RW contributed equally to this work, they contributed to the collection of data, to the analysis of the results. RW revised manuscripts. JZ and PX supported the total work of the YW. RW was in charge of the article writing, communicating with the reviewers and made multiple revisions for final publication. All authors contributed to the article and approved the submitted version.

## Conflict of Interest

The authors declare that the research was conducted in the absence of any commercial or financial relationships that could be construed as a potential conflict of interest.

## Publisher’s Note

All claims expressed in this article are solely those of the authors and do not necessarily represent those of their affiliated organizations, or those of the publisher, the editors and the reviewers. Any product that may be evaluated in this article, or claim that may be made by its manufacturer, is not guaranteed or endorsed by the publisher.
